# An Enhanced Affine Projection Algorithm Based on the Adjustment of Input-Vector Number

**DOI:** 10.3390/e24030431

**Published:** 2022-03-20

**Authors:** Jaewook Shin, Jeesu Kim, Tae-Kyoung Kim, Jinwoo Yoo

**Affiliations:** 1 Department of Electronic Engineering, Kumoh National Institute of Technology, Gumi 39177, Korea; shinshingo@kumoh.ac.kr; 2 Department of Cogno-Mechatronics Engineering, Pusan National University, Busan 46241, Korea; jeesukim@pusan.ac.kr; 3 Department of Optics and Mechatronics Engineering, Pusan National University, Busan 46241, Korea; 4 Department of Electronic Engineering, Gachon University, Seongnam 13120, Korea; tkkim@gachon.ac.kr; 5 Department of Automobile and IT Convergence, Kookmin University, Seoul 02707, Korea

**Keywords:** adaptive filter, affine projection algorithm, input-vector number, adjustment, convergence rate, steady-state estimation error, filter performance

## Abstract

An enhanced affine projection algorithm (APA) is proposed to improve the filter performance in aspects of convergence rate and steady-state estimation error, since the adjustment of the input-vector number can be an effective way to increase the convergence rate and to decrease the steady-state estimation error at the same time. In this proposed algorithm, the input-vector number of APA is adjusted reasonably at every iteration by comparing the averages of the accumulated squared errors. Although the conventional APA has the constraint that the input-vector number should be integer, the proposed APA relaxes that integer-constraint through a pseudo-fractional method. Since the input-vector number can be updated at every iteration more precisely based on the pseudo-fractional method, the filter performance of the proposed APA can be improved. According to our simulation results, it is demonstrated that the proposed APA has a smaller steady-state estimation error compared to the existing APA-type filters in various scenarios.

## 1. Introduction

**Figure 1:**
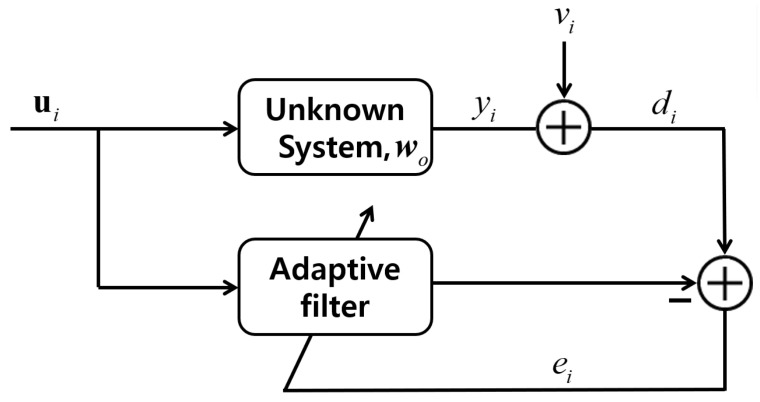
Structure of the adaptive filter.

Many representative algorithms based on the adaptive filtering theory have been successfully applied in channel estimation, system identification, and noise/echo cancellation [1,2,3,4,5,6,7,8,9,10,11,12,13]. As can be seen in Figure 1, the main purpose of an adaptive filter is the accomplishment for the precise estimation of filter coefficients, to minimize error signals with the same input signals. The least-mean squares (LMS) and normalized least-mean squares (NLMS) algorithms are the representative adaptive filtering algorithms because of easy implementation and low complexity. Compared to the LMS and NLMS algorithms [9], the affine projection algorithm (APA) [7] exhibits a high convergence rate for highly correlated input data because it employs multiple input vectors instead of only one input vector. However, the APA is computationally complex and incurs a large steady-state estimation error. Notably, a high input-vector number leads to rapid convergence but a large estimation error. In contrast, a low input-vector number leads to slow convergence but a small estimation error. Therefore, by adjusting the input-vector number, a high convergence rate and small steady-state estimation error can likely be simultaneously realized.

Recently, several researchers have focused on the input-vector number to enhance the performance of APAs, with several representative algorithms being the APA with dynamic selection of input vectors (DS-APA) [14], APA with selective regressors (SR-APA) [15], and APA with evolving order (E-APA) [16]. Although these algorithms outperform the conventional APA by achieving smaller estimation errors, the convergence rate and steady-state estimation error remain to be optimized.

Considering these aspects, in this study, a novel variable input-vector number APA framework is developed, in which the input-vector number is modified using a pseudo-fractional method based on the concept of the pseudo-fractional tap length [17] to ensure small steady-state estimation errors. The pseudo-fractional method employs the integer and fractional input-vector number and relaxes the constraint of the conventional APA that requires the input-vector number to be integer. Thus, the input-vector number for the proposed APA can be increased or decreased by comparing the averages of the accumulated errors. Compared to the existing algorithms, such as the conventional APA, DS-APA, SR-APA, and E-APA, the proposed algorithm based on the pseudo-fractional method achieves a higher convergence rate and smaller steady-state estimation error.

This paper is organized as follows. In the following Section 2, the conventional APA is explained. In Section 3, the proposed APA based on the adjustment of input-vector number by using the pseudo-fractional strategy is explained in detail. In Section 4, we present the simulation results to verify the performance of the proposed APA. Finally, Section 5 gives the conclusions of this paper.

## 2. Conventional Affine Projection Algorithm

Consider reference data $d_{i}$ obtained from an unknown system

(1) $$\begin{array}{c} d_{i}=u_{i}^{T}w_{o}+v_{i}, \end{array}$$

where $w_{o}$ is the n-dimensional column vector of the unknown system, which must be estimated; $v_{i}$ indicates the measurement noise, with variance $\sigma_{v}^{2}$; and $u_{i}$ denotes an n-dimensional column input vector, $u_{i}={\left[u_{i}\;\;u_{i-1}\;\;\ldots \;\;u_{i-n+1}\right]}^{T}$. The conventional APA is derived by minimizing the $L_{2}$-norm of the difference of filter coefficient vectors between at iteration *i* and i+1 with setting a posteriori the error vector as zero, as follows:

(2) $$\min_{{\hat{w}}_{i+1}}\;||{\hat{w}}_{i+1}-{\hat{w}}_{i}{\left|\right|}_{2}^{2}\;\mathrm{subject}\;\mathrm{to}\;d_{i}=U_{i}^{T}{\hat{w}}_{i+1},$$

where $e_{i}=d_{i}-U_{i}^{T}{\hat{w}}_{i}$, ${\hat{w}}_{i}$ is the estimate of $w_{o}$ at iteration *i*, μ is the step-size parameter, *M* is the input-vector number that means the number of current input vectors used for the APA update, and

(3) $$\begin{array}{c} U_{i}=\left[u_{i}\;\;u_{i-1}\;\;\ldots \;\;u_{i-M+1}\right],\;d_{i}={\left[d_{i}\;\;d_{i-1}\;\;\ldots \;\;d_{i-M+1}\right]}^{T}. \end{array}$$

Through the Lagrange multiplier and gradient descent method, the filter update equation of the conventional APA can be presented as [7]

(4) $$\begin{array}{c} {\hat{w}}_{i+1}={\hat{w}}_{i}+\mu U_{i}{\left(U_{i}^{T}U_{i}+\beta I\right)}^{-1}e_{i}, \end{array}$$

where β is the regularization parameter that is usually a very small positive value, and I denotes the identity matrix.

## 3. Enhanced Affine Projection Algorithm Based on the Adjustment of Input-Vector Number

The conventional APA has the constraint that the input-vector number of APA should be integer. In this context, the filter performance can be enhanced if the input-vector number can include both integer and non-integer parts. Therefore, we develop a novel APA by the adjustment of the input-vector number using a pseudo-fractional method that is based on the concept of the pseudo-fractional tap length [17]. The pseudo-fractional method adopts the integer and fractional input-vector number by relaxing the integer constraint for the input-vector number. The integer input-vector number refers to the integer part of the fractional input-vector number when the difference in the integer and fractional input-vector number is larger than a predetermined value. In this proposed framework, the input-vector numbers are dynamically adjusted to enhance the performance of the proposed algorithm in terms of the convergence rate and steady-state estimation error. Moreover, the leaky factor is implemented in the adaptation rule of the fractional input-vector number.

According to the adaptation rule, as defined in Equation (5), the integer input-vector number remains constant until the change in the fractional input-vector number accumulates to a certain extent. $P_{i}$ is the pseudo-fractional input-vector number, which can be assigned positive integer values.

(5) $$\begin{array}{c} P_{i+1}=\left\{\begin{array}{cc} \; & \left(P_{i}-\alpha \right)-\gamma \left(AASE_{M_{i}}\left(i\right)-AASE_{M_{i}-1}\left(i\right)\right),\;\mathrm{if}M_{i}⩾2\\ \; & \left(P_{i}-\alpha \right)-\gamma \left(AASE_{M_{i}+1}\left(i\right)-AASE_{M_{i}}\left(i\right)\right),\;\mathrm{otherwise}. \end{array}\right. \end{array}$$

In this expression, α and γ are small positive numbers, α is the leaky factor that satisfies α≪γ, and $M_{i}$ is the integer input-vector number at instant *i*. *AASE*, which is average of the accumulated squared errors, is defined as

(6) $$\begin{array}{c} AASE{}_{M}\left(i\right)≜\frac{\sum_{N=0}^{M-1}e_{N}^{2}\left(i\right)}{M}. \end{array}$$

Subsequently, the integer input-vector number $M_{i}$ can be determined as

(7) $$\begin{array}{c} M_{i}=\left\{\begin{array}{cc} \; & \max\left\{\min\left\{\left\lfloor P_{i-1}\right\rfloor ,M_{\max}\right\},1\right\},\;\mathrm{if}\;\left|M_{i-1}-P_{i-1}\right|⩾\delta \\ \; & M_{i-1},\;\mathrm{otherwise}. \end{array}\right. \end{array}$$

where the ⌊.⌋ operator approximates the value to the nearest integer, and δ is the threshold parameter. $M_{i}$ is updated to satisfy $1\leq M_{i}\leq M_{\max}$, where $M_{\max}$ is the maximum input-vector number. The threshold parameter δ is set to 0.5 to maximize the filter performance of our proposed APA. The proposed APA is updated using the following expression:

(8) $$\begin{array}{c} {\hat{w}}_{i+1}={\hat{w}}_{i}+\mu U_{i,M_{i}}{\left(U_{i,M_{i}}^{T}U_{i,M_{i}}+\beta I\right)}^{-1}e_{i,M_{i}}. \end{array}$$

where $U_{i,M_{i}}=\left[u_{i}\;\;u_{i-1}\;\;\ldots \;\;u_{i-M_{i}+1}\right]$, $e_{i,M_{i}}={\left[e_{0}\left(i\right)\;\;e_{1}\left(i\right)\;\;\ldots \;\;e_{M_{i}-1}\left(i\right)\right]}^{T}$, and $M_{i}$ is determined according to the adaptation rule for the fractional input-vector number. Through the above-mentioned method, the proposed APA can accomplish the variable input-vector number strategy to enhance the filter performance in terms of the convergence rate and steady-state estimation error.

Because the proposed algorithm is designed for stationary environments, the input-vector number must be re-initialized to achieve a high tracking performance each time the target system is changed. To this end, the re-initialization method reported in [18] is used as a reference and modified, as illustrated in Algorithm 1.

**Algorithm 1:** Re-initialization of the input-vector number. $e_{th}≜\mu \sigma_{v}^{2}M_{max}/\left(2-\mu \right)$, flag =0, $e_{avg}=e_{0}^{2}$, $\lambda ,\alpha_{1},\alpha_{2}$: user defined. **for** each *i* **do**         **if** ($e_{i}^{2}<\alpha_{1}*e_{th}$)                flag =1         **else if** ( flag = 1 and $\alpha_{2}*e_{avg}<e_{i}^{2}$ )               flag =0, $e_{avg}=e_{i}^{2}$, $M_{i}=M_{\max}$, $P_{i}=M_{\max}$         **end if**         $e_{avg}=\lambda e_{avg}+\left(1-\lambda \right)e_{i}^{2}$ **end for**


## 4. Experimental Results

The performance of the proposed algorithm is evaluated considering a channel estimation framework. The channel of the unknown system is generated using a moving average model with 16 taps (n=16). The adaptive filter and unknown channel are assumed to have the same number of taps. Moreover, the noise variance ${\sigma_{v}}^{2}$ is assumed to be known a priori, as its value can be estimated during silences in many practical applications [19,20,21]. The input signal $u_{i}$ is generated by filtering a white, zero-mean Gaussian random sequence by using the following system:

(9) $$\begin{array}{ccc} G_{1}\left(z\right) & =\frac{1}{1-0.9z^{-1}},\;\;G_{2}\left(z\right) & =\frac{1+0.6z^{-1}}{1+z^{-1}+0.21z^{-2}}. \end{array}$$

The measurement noise $v_{i}$ is added to $y_{i}$ with a signal-to-noise ratio (SNR) of 30 dB. In this case, the SNR is defined by $10\log_{10}\left(E\left[y_{i}^{2}\right]/E\left[\upsilon_{i}^{2}\right]\right)$ and $y_{i}=u_{i}^{T}w_{o}$. $P_{0}$ and $M_{0}$ are set to $M_{\max}$, which is the initial input-vector number of the proposed APA. The mean squared deviation (MSD), i.e., $E∥w_{o}-{\hat{w}}_{i}{∥}^{2}$, is adopted as the indicator for the algorithm performance. The simulation results are obtained through ensemble averaging over 100 independent trials, and the input signals are generated using $G_{1}\left(z\right)$ and $G_{2}\left(z\right)$. Furthermore, to examine the tracking performance of the proposed algorithm, the coefficients of the unknown filter taps are abruptly varied at time $5\times 10^{3}$ in the simulation. The parameters of the proposed algorithm are set as follows: $M_{\max}$ = 8, μ = 0.1, α = 0.03, γ=1−α, and β=0.000001. The re-initialization parameters are set as λ = 0.95, $\alpha_{1}$ = 10, $\alpha_{2}$ = 40 [18].

### 4.1. System Identification Verification for Correlated Input

**Figure 2:**
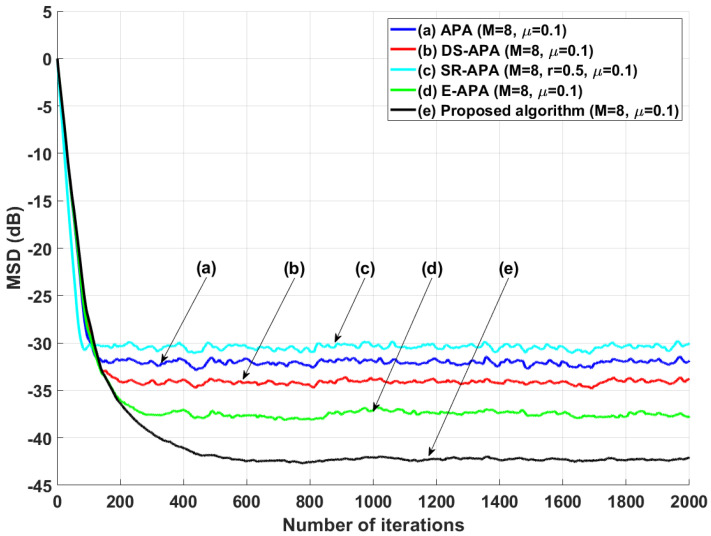
MSD learning curves of the conventional APA, DS-APA, SR-APA, E-APA, and proposed APA when the input signals are generated using $G_{1}\left(z\right)$, with *n* = 16 and SNR = 30 dB.

**Figure 3:**
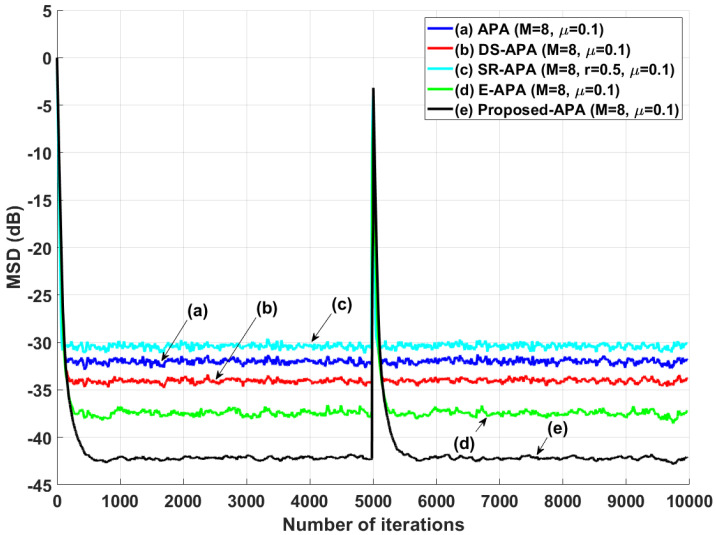
MSD learning curves of the conventional APA, DS-APA, SR-APA, E-APA, and proposed APA when the unknown system is abruptly changed from $w_{o}$ to $-w_{o}$ at iteration $5\times 10^{3}$; the input signals are generated using $G_{1}\left(z\right)$, with *n* = 16 and SNR = 30 dB.

**Figure 4:**
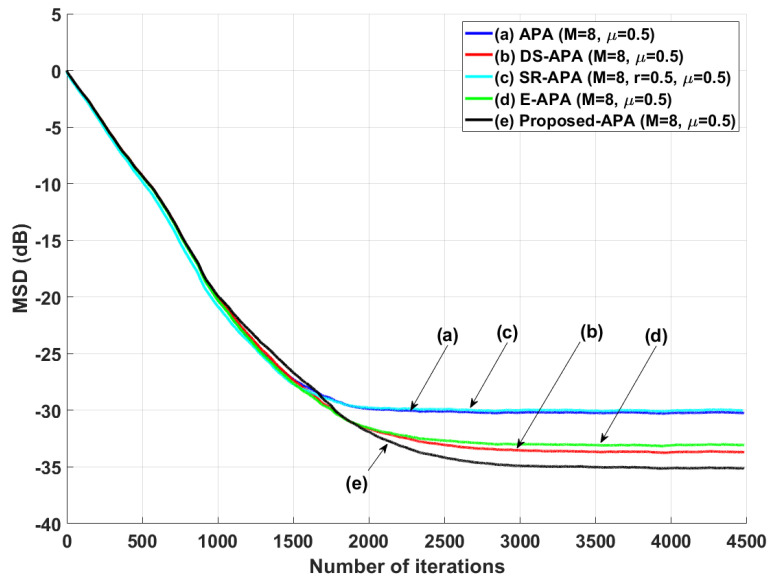
MSD learning curves of the conventional APA, DS-APA, SR-APA, E-APA, and proposed APA when the input signals are generated using $G_{1}\left(z\right)$, with *n* = 256 and SNR = 30 dB.

**Figure 5:**
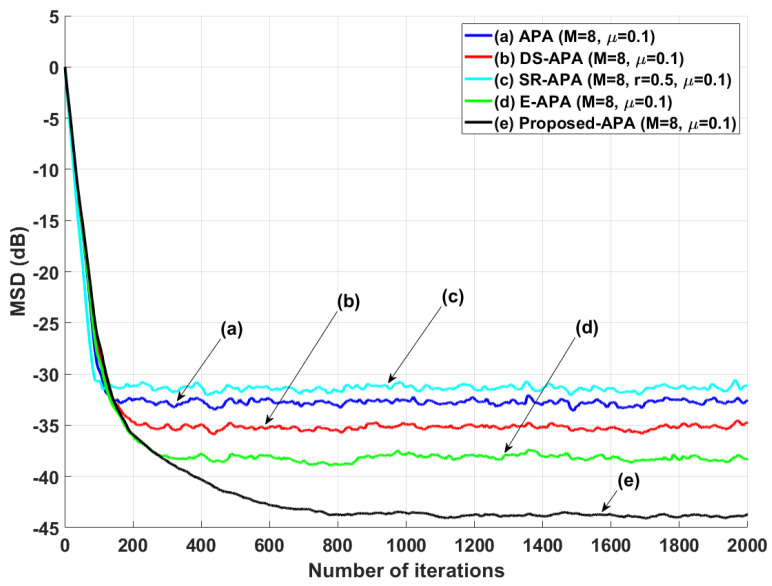
MSD learning curves of the conventional APA, DS-APA, SR-APA, E-APA, and proposed APA when the input signals are generated using $G_{2}\left(z\right)$, with *n* = 16 and SNR = 30 dB.

**Figure 6:**
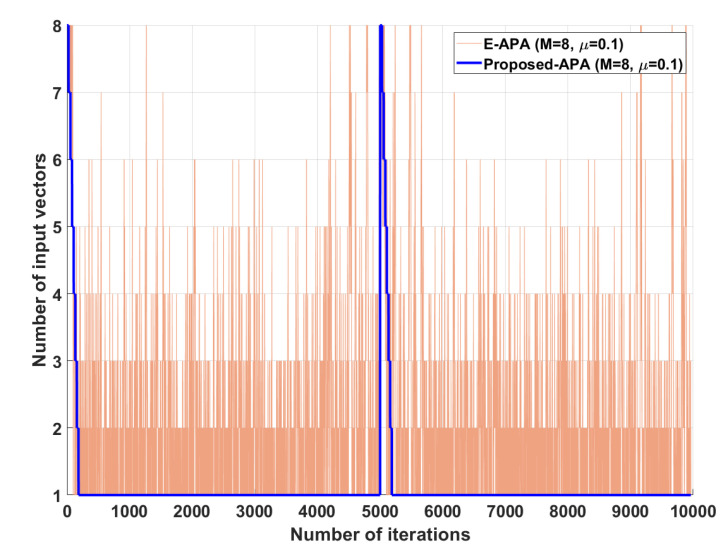
Comparison of the input-vector numbers over one trial for the E-APA and proposed APA when the input signals are generated using $G_{1}\left(z\right)$, with *n* = 16 and SNR = 30 dB.

**Figure 7:**
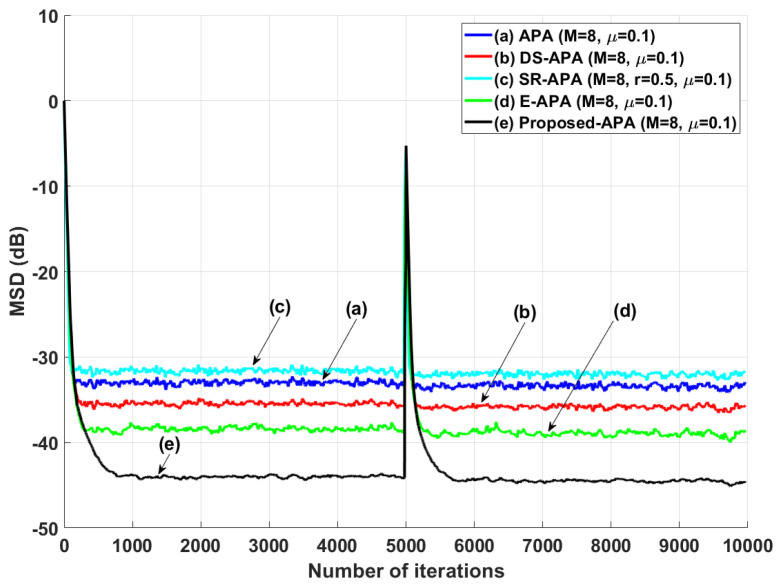
MSD learning curves of the conventional APA, DS-APA, SR-APA, E-APA, and proposed APA when the unknown system is abruptly changed from $w_{o}$ to $-w_{o}$ at iteration $5\times 10^{3}$; the input signals are generated using $G_{2}\left(z\right)$, with *n* = 16 and SNR = 30 dB.

Figure 2 shows the MSD learning curves of the conventional APA [7], DS-APA [14], SR-APA [15], E-APA [16], and proposed APA when the input vector is generated using $G_{1}\left(z\right)$ and the AR input model. As can be seen in Figure 2, the proposed APA has the smaller steady-state estimation error as compared to the existing APA-type algorithms. In addition, even though the system change occurs suddenly as shown in Figure 3, the proposed algorithm maintains the filter performance in aspects of the convergence rate and steady-state estimation error. Moreover, when the tap length is long (n=256), the proposed APA also has the smaller steady-state estimation errors, as shown in Figure 4. Figure 5 also shows the MSD learning curves of the conventional APA, DS-APA, SR-APA, E-APA, and proposed APA when the input vector is generated using $G_{2}\left(z\right)$ and the ARMA input model. As can be seen in Figure 5, the proposed APA outperforms the existing algorithm in terms of the convergence rate and steady-state estimation error. Figure 6 represents the comparison of input vector numbers over one trial for the E-APA and proposed-APA. According to Figure 6, it can be verified that the proposed APA uses the input-vector number efficiently. Figure 7 shows the tracking performance of the existing and proposed algorithms when the unknown system is abruptly changed from $w_{o}$ to $-w_{o}$. As can be seen in Figure 3 and Figure 7, the tracking performance of the proposed algorithm is maintained without any degradation in the convergence rate or steady-state estimation errors, owing to the use of the re-initialization method [18]. These simulation results demonstrate that the proposed variable input-vector number APA achieves a smaller steady-state estimation error than the existing algorithms.

**Figure 8:**
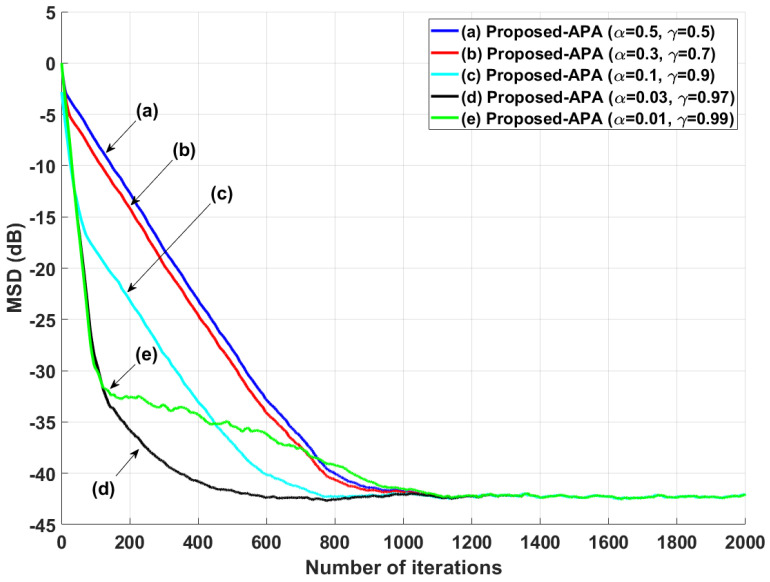
MSD learning curves of the proposed APAs with several values of α and γ when the input signals are generated using $G_{1}\left(z\right)$, with *n* = 16 and SNR = 30 dB.

In our proposed APA, it is important to decide on the values of α and γ=1−α in Equation (5), because the parameters α and γ are dominant factors to determine the input-vector number. Therefore, we investigate the filter performance comparison according to several values of α and γ. As shown in Figure 8, the proposed APA has the best performance in case of α=0.03 with γ=1−α. Even though the parameter tuning method cannot precisely provide an optimal value, α=0.03 gives the best performance in our simulation scenarios. Since the parameter α and γ values have a restriction between 0 and 1, it is easy to find the proper α and γ values based on the variation of α and γ values to ensure the improved filter performance in each scenario.

**Figure 9:**
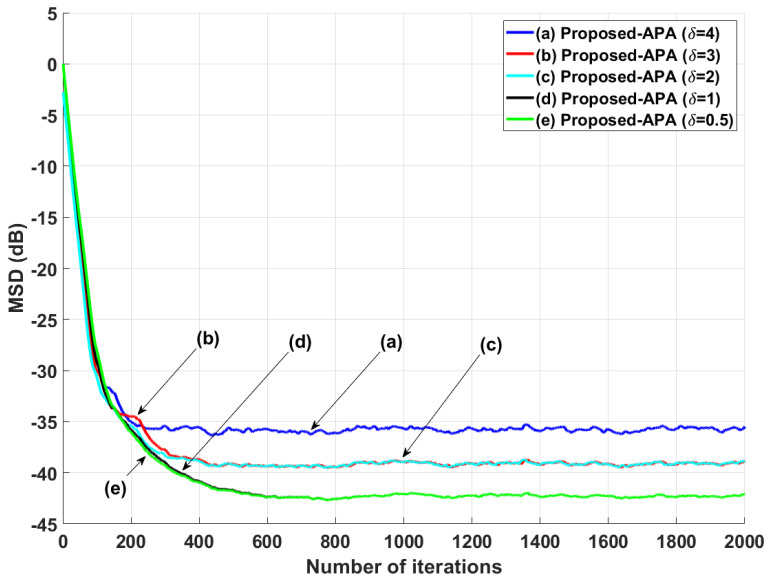
MSD learning curves of the proposed APAs with several values of δ when the input signals are generated using $G_{1}\left(z\right)$, with *n* = 16 and SNR = 30 dB.

Moreover, the parameter δ in Equation (7) is also a dominant factor to decide on the filter performance of the proposed APA. To verify the influence of the parameter δ, the comparison simulation according to the variation of δ is shown in Figure 9. Since the filter performance of the proposed APA can be maximized experimentally when the parameter δ is set to 0.5, the proposed APA uses δ=0.5 consistently in all simulations.

### 4.2. Speech Input Verification Including a Double-Talk Situation

**Figure 10:**
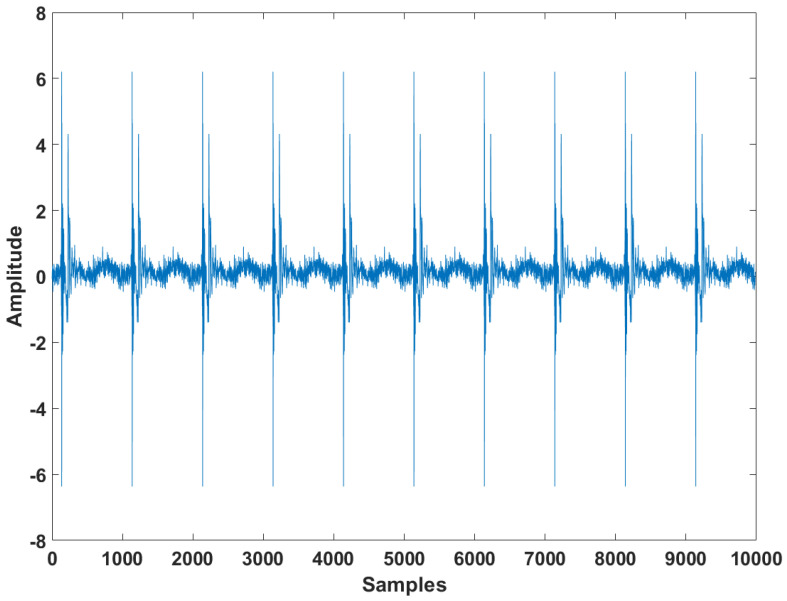
Speech input signals.

**Figure 11:**
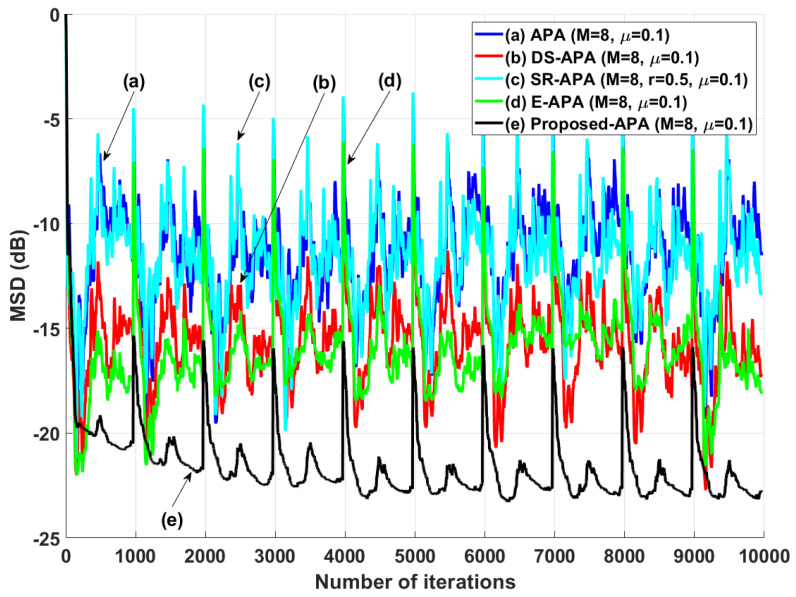
MSD learning curves for the speech input.

**Figure 12:**
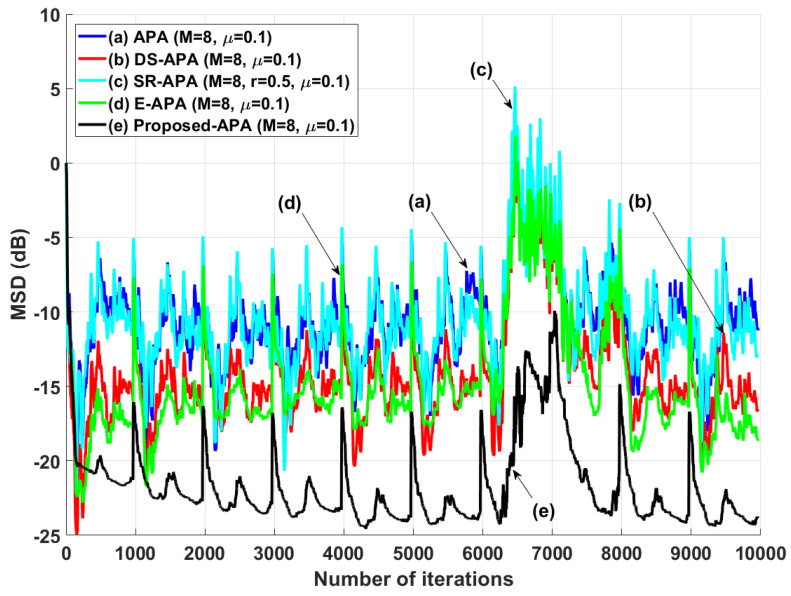
MSD learning curves for the double-talk situation.

The proposed APA was experimented by using the speech input signals to ensure the filter performance in practical scenarios as can be seen in Figure 10. Since the speech input signals are real human speech data, this simulation result for speech input increases the reliability of the proposed APA for practical use. As can be seen in Figure 11, we can find that the proposed APA can accomplish the smaller steady-state estimation errors compared to the other algorithms. The proposed algorithm was also tested in a double-talk situation as shown in Figure 12. The far-end input signal and near-end input signal were speech signals where the power of the near-end input signal was 2 times greater than that of the far-end input signal. The near-end input signal was added between iterations $6.2\times 10^{3}$ and $7.2\times 10^{3}$. Figure 12 shows that the proposed APSA delivered better performance than the other algorithms in terms of the convergence rate and the steady-state estimation error. Even after the double-talk occurrence, we can find that the proposed APSA consistently has smaller steady-state estimation errors. As can be seen in Figure 11 and Figure 12, the proposed APA can achieve the better filter performance in aspects of the convergence rate and steady-state estimation error in harsh speech-input scenarios.

### 4.3. Comparison for Computational Complexity

**Figure 13:**
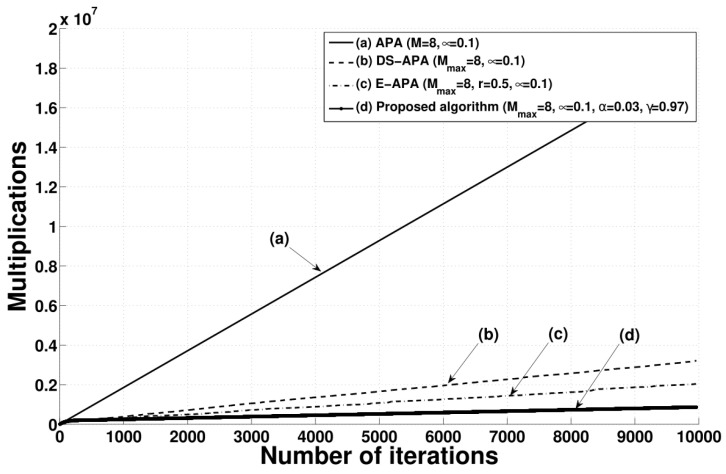
Accumulated sum of multiplications for the conventional APA, DS-APA, E-APA, and proposed algorithm.

Table 1 presents the iteration-wise computational complexity values of the conventional APA, DS-APA, E-APA, and proposed algorithm, for which the input-vector numbers are *M*, $M_{j}$, $M_{k}$, and $M_{i}$, respectively. Figure 13 shows the accumulated sum for the multiplications of the proposed APA compared to the existing APA-type algorithms. As can be seen in Figure 13, the proposed algorithm can be executed with smaller computational complexity than the other algorithms. The values for the proposed algorithm are considerably smaller than those of the conventional APA, DS-APA, and E-APA owing to the considerably smaller input-vector numbers of the proposed algorithm in the steady state.

## 5. Conclusions

This paper proposed a novel APA based on the adjustment of input-vector number to enhance the filter performance in aspects of both convergence rate and steady-state estimation error. In this framework, the input-vector number for the proposed APA was determined using the pseudo-fractional method. Because the pseudo-fractional method relaxed the constraint of the conventional APA that required the input-vector number to be integer, it was meaningful to decide the input-vector number more precisely to improve the filter performance. Specifically, the proposed method dynamically adjusted the input-vector number by using the proposed adaptation rule for the fractional input-vector number. According to the adaptation rule, the current projection order was set by comparing the *AASE* values reasonably. Simulation results with system identification and speech input scenarios demonstrated that the proposed APA had smaller steady-state estimation error as compared to the existing APA-type algorithms. Moreover, we plan to develop an improved APA to adjust both the projection order and step size based on the proposed method. Because the E-APA [16] was expanded by using the variable projection order and step-size concept simultaneously to enhance the filter performance in aspects of convergence rate and steady-state estimation error, it can be an effective way for our proposed APA to improve the filter performance. By extension, we will research the expansion for various kinds of adaptive filter through the variable projection-order and step-size strategies to improve the filter performance.

## Figures and Tables

**Table 1:** Computational complexity of conventional APA, DS-APA, E-APA, and proposed APA.

	APA	DS-APA	E-APA	Proposed APA
Input-Vector				
Number	*M*	$M_{j}$	$M_{k}$	$M_{i}$
#(×/÷)	$(M^{2}+2M)n$	$(M_{j}^{2}+M_{j}+M)n$	$(M_{k}^{2}+2M_{k})n+$	$(M_{i}^{2}+2M_{i})n+$
	$+M^{3}+M^{2}$	$+M_{j}^{3}+M_{j}^{2}$	$M_{k}^{3}+M_{k}^{2}$	$M_{i}^{3}+M_{i}^{2}$
	$+M^{3}+M^{2}$	$+M_{j}^{3}+M_{j}^{2}$	$+M_{k}+1$	$+M_{i}+2$
#(comparisons)	0	*M*	2	2

## Data Availability

Not applicable.
